# Direct economic burden attributable to age-related diseases in China: An econometric modelling study

**DOI:** 10.7189/jogh.13.04042

**Published:** 2023-05-05

**Authors:** Xin Ye, Ming Wang, Yiqi Xia, Ping He, Xiaoying Zheng

**Affiliations:** 1 Institute for Global Public Policy, Fudan University, Shanghai, China; 2 LSE-Fudan Research Center for Global Public Policy, Fudan University, Shanghai, China; 3 School of Public Health, Peking University, Beijing, China; 4 China Center for Health Development Studies, Peking University, Beijing, China; 5 School of Population Medicine and Public Health, Chinese Academy of Medical Sciences / Peking Union Medical College, Beijing, China

## Abstract

**Background:**

Aging is a strong risk factor for many chronic diseases. However, the economic burden attributable to age-related diseases remains unclear. We aimed to calculate the economic burden attributable to age-related diseases in China.

**Methods:**

We used an econometric modelling approach from the China Health and Retirement Longitudinal Survey (CHARLS), which is based on a longitudinal observational data set from middle-aged and older adults aged 45+ in 2011, 2013, and 2015.

**Results:**

We calculated the total direct economic burden attributable to age-related diseases for outpatient and inpatient services among adults aged 45 and above in China, which was approximately 288.368 billion US dollars (US$), US$379.901 billion, and US$616.809 billion in 2011, 2013, and 2015, respectively, taking up 19.48%, 21.11% and 32.03% of the overall health care expenses in the same year. The proportion of dyslipidemia was the largest, followed by hypertension in all the three years; hearing problems accounted for the lowest proportion.

**Conclusions:**

The alarming upward trend in age-related economic burden in China calls for urgent interventions to prevent or slow down the accumulation of damage associated with age-related diseases.

Not only developed countries, but many developing countries including China are facing an unprecedented and rapidly growing elderly population [1]. By 2021, China has achieved its first centenary goal of building a moderately prosperous society in all respects, with average life expectancy rising from 70.1 years in 1996 to 77.3 years in 2019 [2]. According to China’s second national census, the number of population aged 60 and above in the mid-1960s was about 38.17 million (5.5% of the total population) [3]. While in 2020, the number of people aged 60 years and above increased seven-fold to 264.02 million (18.70% of the total population), and the number of people aged 65 years and above was 190.64 million (13.50%) [4]. China was expected to become an aged society by 2022 and a super-aged society by 2033 [5].

An aging population is accompanied by an increasing burden of non-communicable diseases (NCDs). Aging is a recognized risk factor for the development of multiple NCDs, including cardiovascular diseases, stroke, cancer, osteoarthritis, and dementia, etc. [6]. Because of the combination of risk factors such as diet, tobacco, etc. in the elderly, the incidence of NCDs has been increasing rapidly [7]. NCDs have high rates of mortality and disability, and require expensive hospitalization expenses [8]. Hospitalization rates in China are even higher than that in the Organization for Economic Co-operation and Development (OECD) countries (about 18%), and the associated high medical expenses bring a huge burden to the whole society and individuals [8,9]. Non-communicable diseases will also put at risk the financial sustainability of all public health care systems [9].

Available research on the exact economic burden attributable to age-related diseases is rather limited. An Italian study using accurate clinical and drug prescription data from Health Search CSD-LPD between 2005 and 2014 found that age-related disease burden accounted for about 20% of the public health care budget every year [10]. Age-related diseases can increase economic burden through mortality, early retirement [11], and reduced productivity [12]. Also, current interventions related to NCDs, including medical treatment and prevention, require a substantial amount of resources [13]. Additionally, lower productivity and reduced labour supply, combined with higher health care expenses, will lead to a decline in total income, further increasing the economic burden [13]. The increased burden of chronic disease is particularly severe in low-income and middle-income countries, which can least afford a health-related backsliding in development [9].

Despite the high prevalence of age-related diseases, little is known about the exact economic burden attributable to age-related diseases in China. An important improvement over existing literature is proposed in this study, which uses nationally representative survey from 2011 to 2015 to calculate the direct economic burden attributable to age-related diseases in China using an econometric modelling. To our knowledge, this is one of the largest longitudinal population samples ever used in this diseases burden calculation, and it can provide evidence of potential economic savings or cost-effective interventions for age-related diseases. There is a need for further investment in interventions aimed at alleviating age-related diseases, as it will contribute to expressively reducing expenditures on the public health care system and improving the quality of life and health of the Chinese population.

## METHODS

### Data and sample

The main data used is from the China Health and Retirement Longitudinal Survey (CHARLS), which aims to collect high-quality data set representing households and individuals aged 45 and older in China and to address the challenges associated with population aging in China [14]. This is a long-term follow-up project, observing the life trajectory and changes of the interviewees. CHARLS combines detailed socioeconomic data with high-quality data on individuals’ physical and mental health. The sample was stratified by urban / rural areas and by gross domestic product (GDP) using probability proportional to size (PPS) to ensure that the sample is representative of the Chinese population, and the data are collected using standardized protocols to ensure consistency across respondents. In addition, survey weights were further taken into account in our estimates to correct for nonresponse and sampling-frame errors and to ensure the representativeness of the sample.

The baseline survey was conducted in 2011 and follow-up surveys were conducted in 2013 and 2015. CHARLS 2011 covered 28 provincial-level units, 150 county-level units, 450 village-level units, 10 257 households and 17 708 samples, representing the middle-aged and older population in China as a whole. In 2013, the project completed the first follow-up interview, and the follow-up rate reached 88%. New individuals were also being added to the CHARLS cohort at each survey wave. In the second follow-up survey in 2015, a total of 11 797 households and 20 284 people were interviewed, with a follow-up rate of 87%. The number of population by sex and age in 2011, 2013 and 2015 are from the China Statistical Yearbooks and China Demographic and Employment Statistical Yearbooks for the corresponding year. The results for China did not include Hong Kong and Macao special administrative regions.

### Variables

#### Age-related diseases

The selection of age-related diseases was based on both the existing literature and the types of age-related diseases covered in the CHARLS questionnaire. Age-related diseases include hearing problems, vision problems, hypertension, dyslipidemia, heart diseases, stroke, lung diseases, asthma, digestive diseases, liver diseases, arthritis, kidney diseases, cancer, diabetes, etc. [15]. In CHARLS 2011, 2013 and 2015, age-related diseases were identified through self-reporting. Participants were asked questions of whether they had these age-related diseases by trained investigators through face-to-face interviews.

#### Healthcare probability and direct economic burden

Healthcare probability and related economic burden were used to calculate direct economic burden attributable to age-related diseases. Healthcare probability included participants’ probability of using inpatient services last month or using outpatient services last year. Direct economic burden attributable to age-related diseases referred to the health care expenditure incurred from having age-related diseases by the patient in the health and non-health sectors during the period of illness. In the survey, respondents were also asked to separately report health care expenditure for each category: monthly outpatient expenses, annual inpatient expenses (only include the fees paid to the hospital), as well as outpatient transportation expenses, inpatient transportation and accommodation expenses incurred by accompanying patients. These expenses included both out-of-pocket health care expenses paid by survey participants and expenses paid by private and public health insurers.

#### Control variables

Control variables were presented as the following: (1) sociodemographic characteristics, including age (continuous), sex (female, male), education (illiterate, primary school, junior middle school, senior middle school and above), registered residents (rural, urban), marital status (single, partnered); (2) health status, including activities of daily living ((ADLs) impaired or unimpaired), instrumental activities of daily living ((IADLs) impaired or unimpaired), depressive symptoms measured by the 10-item Center for Epidemiological Studies Depression Scale (CES-D-10) scores (yes, no); (3) health behaviours, including current smoking (yes, no), current drinking (yes, no).

### Statistical analysis

This analysis followed previous literature to focus on all-cause health care spending [16,17], because age-related diseases could result in deterioration of overall physical and mental health outcomes. An econometric modelling was used to compare the annual per capita direct economic burden of individuals with and without age-related diseases, thus obtaining the direct economic burden of age-related diseases.

#### Age-related Diseases-attributable Fraction through the econometric modelling (*ADAF^EC^*)

The regression applied multiple mixed models based on the panel data of CHARLS 2011, 2013 and 2015. As age-related diseases can gradually progress over time, we used mixed models to account for the dynamic longitudinal association between age-related diseases and health care utilization [18,19]. Mixed models took into account within-subject correlation of time-varying age-related diseases and health care utilization over four years of follow-up (two waves).

The first step was the selection model, and the mixed logistic regression model was used to estimate the probability of using health care services. The specific regression model is as follows:

*DExp_itx_ = γ*_1_*ARD_itk_ + γ*_2_*X_itk_ + γ*_3_*Y_itk_ + γ*_4_*Z_itk_ + α_ik_ + υ_k_ + ω_t_*∙∙∙∙∙∙ (1)

The second step was to estimate the direct economic burden by mixed linear regression model on log (positive) expenditure:

ln *Exp_itk_ = β*_1_*ARD_itk_ + β*_2_*X_itk_ + β*_3_*Y_itk_ + β*_4_*Z_itk_ + α’_ix_ + υ’_k_ + ω’_t_*∙∙∙∙∙∙ (2)

Where *DExp_itk_* is a dummy variable, indicating whether individual *i* in the community *k* uses health care services (including inpatient and outpatient treatment) in the year ; ln *Exp_itk_* represents the natural log of self-reported overall health care expenditures of individual *i* in the community *k* in the year *t* (not specific to age-related diseases); *ARD_itk_* is the status of age-related diseases; *X_itk_* are sociodemographic characteristics; *Y_itk_* are the health variables of ADLs, IADLs, and depressive symptoms; *Z_itk_* are health behaviours of smoking and drinking. υ*_k_*、υ'*_k_* are community-level variance; ω*_t_*、ω'*_t_* are time variance.

After obtaining the parameters estimated by the above two equations, we applied them to the following formula to calculate the Age-related Diseases-attributable Fraction (*ADAF^EC^*) based on the econometric modelling by inpatient and outpatient services, marked as *ADAF_υ_^EC^* and *ADAF_h_^EC^*, respectively. The estimates were to predict the share of the annual health care spending that would be reduced if people had no age-related diseases. The attributable fraction was calculated by dividing the total age-related diseases-attributable health care spending by the total predicted spending for the entire population. The former was projected by subtracting the predicted health care spending for those with age-related diseases from their predicted spending had they had no age-related diseases.



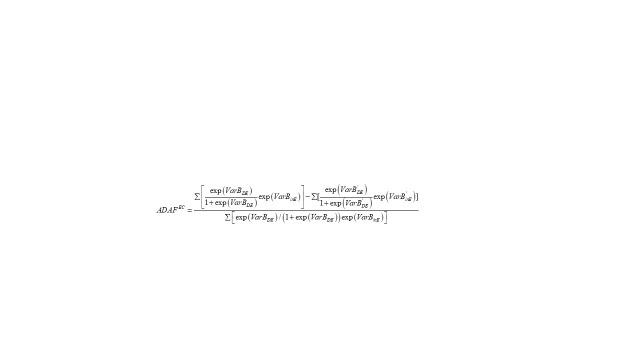

(3)


Where *Var* are individual-level variables; *B* is the regression parameter of the “actual population”; *B’* is the regression parameter of the “hypothetical population”. The “factual population” refers to the actual survey objects, and the “hypothetical population” refers to the population under the assumption that all people have no age-related diseases.

#### Direct Economic Burden (*DEB^EC^*)

To obtain the average Direct Economic Burden (*DEB^EC^*) over three years, we first multiplied *ADAF^EC^* and the direct economic burden of each category of medical services, then summed the values of all groups. Then we calculated the economic burden per person per year from the CHARLS survey, and multiplied by the number of middle-aged and older adults in China, so as to extrapolate cost estimates from the CHARLS survey to all individuals aged 45 or over in China. The formula is as follows:

*DEB^EC^* = (*PV_t_* + *PVN_t_*) × *QV_t_* × *POP_t_* × *ADAF_v_^EC^* + (*PH_t_* + *PHN_t_*) × *QH_t_* × *POP_t_* × *ADAF_h_^EC^*∙∙∙∙∙∙∙∙∙∙ (4)

Where *PV* is the medical cost per outpatient visit; *PVN* is the transportation cost per outpatient visit; *QV* is the average number of outpatient visits per person per year; *PH* is the medical cost per hospital stay; *PHN* is the average cost of transportation, nutrition, and nursing expenses per hospital stay; *QH* is the average number of hospitalizations per person per year; *POP* is the number of population aged 45 years and above in the year; *t* is the year 2011, 2013 and 2015. All monetary amounts were adjusted to 2015 dollars using the health care component of the Consumer Price Index (CPI) provided by the National Bureau of Statistics [20].

## RESULTS

**Table 1** reports the characteristics of respondents from the CHARLS 2011-2015. Among the respondents, 20.25% reported having age-related diseases, and they might be more likely to be older (*P* < 0.001), female (*P* < 0.001), less-educated (*P* < 0.001), single (*P* < 0.001), smoking (*P* = 0.046), drinking (*P* < 0.001), having impaired ADLs (*P* < 0.001), impaired IADLs (*P* < 0.001), depressive symptoms (*P* < 0.001), and having outpatient (*P* < 0.001) or inpatient treatment (*P* < 0.001) last year. It was worth noting that, *P*-values in **Table 1** are probably caused by the large sample sizes. Table S1 in the **Online Supplementary Document** presents summary statistics of detailed age-related diseases.

**Table 1:** Summary statistics of study sample, 2011-2015 (n = 32 418)

Characteristics	People without age-related diseases	People with age-related diseases	*P* value
	**n = 6538 (20.25%)**	**n = 25 744 (79.75%)**	
Age, mean (SD)			
*45-59 years old*	52.18 (0.06)	53.20 (0.04)	<0.001
*60+ years old*	66.62 (0.13)	67.82 (0.05)	<0.001
Sex, n (%)			<0.001
*Male*	3160 (48.33%)	10 962 (42.59%)	
*Female*	3378 (51.67%)	14 776 (57.41%)	
Education, n (%)			<0.001
*Illiterate*	2530 (38.70%)	12 616 (49.02%)	
*Primary school*	1372 (20.99%)	5578 (21.67%)	
*Junior high school*	1689 (25.83%)	4932 (19.16%)	
*Senior high school and above*	947 (14.48%)	2612 (10.15%)	
Residency, n (%)			0.137
*Urban*	2400 (36.71%)	9194 (35.72%)	
*Rural*	4138 (63.29%)	16 544 (64.28%)	
Marital status, n (%)			<0.001
*Separated / divorced / widowed*	580 (8.87%)	3569 (13.87%)	
*Married / cohabiting*	5958 (91.13%)	22 169 (86.13%)	
Smoking status, n (%)			<0.001
*Smoking*	1983 (30.33%)	6353 (24.68%)	
*No smoking*	4555 (69.67%)	19 385 (75.32%)	
Drinking status, n (%)			<0.001
*Drinking*	2460 (37.63%)	7832 (30.43%)	
*No drinking*	4078 (62.37%)	17 906 (69.57%)	
ADLs, n (%)			<0.001
*Unimpaired ADLs*	6157 (94.17%)	20 128 (78.20%)	
*Impaired ADLs*	381 (5.83%)	5610 (21.80%)	
IADLs, n (%)			<0.001
*Unimpaired IADLs*	5783 (88.45%)	18 980 (73.74%)	
*Impaired IADLs*	755 (11.55%)	6758 (26.26%)	
Depressive symptoms, n (%)			<0.001
*No depression*	5195 (79.46%)	15 589 (60.57%)	
*Depression*	1343 (20.54%)	10 149 (39.43%)	
Outpatient treatment last year, n (%)			<0.001
*No*	5813 (88.98%)	19 613 (76.28%)	
*Yes*	720 (11.02%)	6099 (23.72%)	
Inpatient treatment last year, n (%)			<0.001
*No*	6225 (95.24%)	22 237 (86.44%)	
*Yes*	311 (4.76%)	3487 (13.56%)	
Outpatient costs, mean (SD)	1880.24 (18447.33)	4360.96 (32943.61)	<0.001
Inpatient costs, mean (SD)	923.92 (7355.83)	2228.09 (11052.93)	<0.001

SD – standard deviation, ADLs – activities of daily living, IADLs – instrumental activities of daily living

### Age-related Diseases-attributable Fraction

Next, *ADAF^EC^* was estimated by age, sex and residency according to the regression results, as shown in **Table 2**. People with dyslipidaemia and hypertension had higher age-related diseases-attributable fraction score, suggesting their close relationship with aging. There was no age-related diseases-attributable fraction to inpatient and outpatient service use for urban male adults aged above 60 years old with hearing problems.

**Table 2:** Age-related Diseases-Attributable Fraction (*ADAF^EC^*) for outpatient and inpatient services among people aged 45 and above in China 2011-2015

Year 2011-2015	Age-related Diseases-Attributable Fraction (*ADAF^EC^*)							
**Male**				**Female**			
	**Urban**		**Rural**		**Urban**		**Rural**	
	**45-59 years old**	**60 years old and above**	**45-59 years old**	**60 years old and above**	**45-59 years old**	**60 years old and above**	**45-59 years old**	**60 years old and above**
Hearing problems								
*Outpatient services*	0.0303	0.0000	0.0374	0.0867	0.0279	0.0691	0.0262	0.0295
*Inpatient services*	0.0061	0.0000	0.0014	0.0437	0.0196	0.0184	0.01156	0.0407
Vision problems								
*Outpatient services*	0.0144	0.0093	0.0209	0.0000	0.0716	0.0153	0.0236	0.0242
*Inpatient services*	0.0117	0.0000	0.0089	0.0128	0.0146	0.0106	0.0114	0.0034
Hypertension								
*Outpatient services*	0.0816	0.0846	0.1031	0.1304	0.1300	0.1083	0.0929	0.1105
*Inpatient services*	0.1691	0.1819	0.0609	0.1277	0.0871	0.1235	0.0636	0.1449
Dyslipidaemia								
*Outpatient services*	0.1564	0.1232	0.0723	0.0558	0.1285	0.1251	0.0792	0.0904
*Inpatient services*	0.1525	0.1799	0.0466	0.0832	0.1175	0.1209	0.0679	0.1005
Heart diseases								
*Outpatient services*	0.0620	0.0594	0.0643	0.1025	0.1047	0.1733	0.1157	0.1343
*Inpatient services*	0.0953	0.2350	0.0545	0.1392	0.1103	0.2028	0.1040	0.1755
Stroke								
*Outpatient services*	0.0141	0.0093	0.0152	0.0069	0.0000	0.0145	0.0028	0.0028
*Inpatient services*	0.0335	0.0802	0.0137	0.0264	0.0063	0.0346	0.0107	0.0234
Lung diseases								
*Outpatient services*	0.0574	0.0197	0.0827	0.1503	0.0891	0.0962	0.0828	0.1355
*Inpatient services*	0.0598	0.1113	0.0368	0.1496	0.0661	0.0829	0.0611	0.1300
Asthma								
*Outpatient services*	0.0084	0.0268	0.0324	0.0723	0.0221	0.0580	0.0019	0.0410
*Inpatient services*	0.0448	0.0499	0.0293	0.0786	0.0432	0.0720	0.0258	0.0738
Digestive diseases								
*Outpatient services*	0.1830	0.1572	0.1377	0.1704	0.2585	0.1448	0.2369	0.2527
*Inpatient services*	0.0724	0.0820	0.0219	0.1008	0.0820	0.0000	0.1202	0.1508
Liver diseases								
*Outpatient services*	0.0765	0.0355	0.0563	0.0484	0.0387	0.0031	0.0446	0.0259
*Inpatient services*	0.0634	0.0536	0.0308	0.0416	0.0696	0.0250	0.0205	0.0423
Arthritis								
*Outpatient services*	0.1956	0.0995	0.1145	0.2088	0.1167	0.2118	0.2144	0.3105
*Inpatient services*	0.0307	-0.0216	0.0676	-0.0312	0.0493	0.0105	0.0834	0.1674
Kidney diseases								
*Outpatient services*	0.0680	0.0400	0.0346	0.0659	0.0510	0.0472	0.0588	0.0508
*Inpatient services*	0.0859	0.0728	0.0162	0.0482	0.0429	0.0491	0.0485	0.0719
Cancer								
*Outpatient services*	0.0096	0.0106	0.0110	0.0184	0.0335	0.0000	0.0130	0.0114
*Inpatient services*	0.0182	0.0287	0.0053	0.0222	0.0211	0.0122	0.0139	0.0209
Diabetes								
*Outpatient services*	0.0586	0.0665	0.0464	0.0240	0.0739	0.0553	0.0509	0.0498
*Inpatient services*	0.0935	0.1440	0.0336	0.0572	0.1023	0.1037	0.0429	0.0668

### Direct Economic Burden attributable to age-related diseases for subgroups

**Table 3** reports DEB (including transportation and accommodation expenses) per capita for outpatient / inpatient services by age, sex, and residency. Per capita direct economic burden was the lowest in 2011, and increased in 2013 and 2015. Direct Economic Burden per capita of older adults aged 60 and above were mostly higher than those of middle-aged adults aged below 60.

**Table 3:** Direct economic burden per capita for outpatient and inpatient services among people aged 45 and above in China 2011-2015 in US dollars (US$)

	Direct economic burden per capita (US$)							
	Male				Female			
	**Urban**		**Rural**		**Urban**		**Rural**	
	**45-59 years old**	**60 years old and above**	**45-59 years old**	**60 years old and above**	**45-59 years old**	**60 years old and above**	**45-59 years old**	**60 years old and above**
Year 2011								
*Outpatient services*	1330	1788	1783	929	3482	2378	1052	1051
*Inpatient services*	1665	2000	1727	1396	1441	1772	1219	1079
Year 2013								
*Outpatient services*	2442	3756	2364	2119	1757	2375	1845	2541
*Inpatient services*	2399	2530	2027	1742	1305	2136	1602	1450
Year 2015								
*Outpatient services*	1928	4288	3183	3448	2705	3307	2565	3356
*Inpatient services*	2522	2885	2569	2210	2639	2500	1916	1714

**Table 4** reports DEB per capita attributable to age-related diseases for outpatient / inpatient services by age, sex, and residency. In general, the DEB per capita attributable to age-related diseases increased year by year in 2011, 2013 and 2015, and economic burden was generally higher for people from urban areas and aged 60 and above.

**Table 4:** Direct economic burden per capita attributable to age-related diseases for outpatient and inpatient services among people aged 45 and above in China 2011-2015 in US dollars (US$)

	Direct economic burden per capita (US$)							
	Male				Female			
	**Urban**		**Rural**		**Urban**		**Rural**	
	**45-59 years old**	**60 years old and above**	**45-59 years old**	**60 years old and above**	**45-59 years old**	**60 years old and above**	**45-59 years old**	**60 years old and above**
Hearing problems								
*Year 2011*	50.4555	0.0000	69.1020	141.5495	125.3914	196.9246	41.6540	74.9198
*Year 2013*	88.6265	0.0000	91.2514	259.8427	74.5983	203.4149	66.8581	133.9745
*Year 2015*	73.8026	0.0000	122.6408	395.5186	127.1939	274.5137	89.3520	168.7618
Vision problems								
*Year 2011*	38.6325	16.6284	52.6350	17.8688	270.3498	55.1666	38.7238	29.1028
*Year 2013*	63.2331	34.9308	67.4479	22.2976	144.8542	58.9791	61.8048	66.4222
*Year 2015*	57.2706	39.8784	89.3888	28.2880	232.2074	77.0971	82.3764	87.0428
Hypertension								
*Year 2011*	390.0795	515.0648	289.0016	299.4108	578.1711	476.3794	175.2592	272.4826
*Year 2013*	604.9381	777.9646	367.1727	498.7710	342.0755	521.0085	273.2877	490.8855
*Year 2015*	583.7950	887.5463	484.6194	731.8362	581.5069	666.8981	360.1461	619.1966
Dyslipidaemia								
*Year 2011*	461.9245	580.0816	209.3891	167.9854	616.7545	511.7226	166.0885	203.4499
*Year 2013*	747.7763	917.8862	265.3754	263.1746	379.1120	555.3549	254.8998	375.4314
*Year 2015*	686.1442	1047.2931	349.8463	376.2704	657.6750	715.9557	333.2444	475.6394
Heart diseases								
*Year 2011*	241.1345	576.2072	208.7684	289.5457	523.5077	771.4690	248.4924	330.5138
*Year 2013*	380.0287	817.6564	262.4767	459.6839	327.8994	844.7683	380.0745	595.7313
*Year 2015*	359.8826	932.6822	344.6774	661.0520	574.2952	1080.1031	496.0345	751.5178
Stroke								
*Year 2011*	74.5305	177.0284	50.7615	43.2645	2.1143	95.7922	15.9889	28.1914
*Year 2013*	114.7987	237.8368	63.7027	60.6099	4.7075	108.3431	22.3074	41.0448
*Year 2015*	111.6718	271.2554	83.5769	82.1352	11.2157	134.4515	27.6832	49.5044
Lung diseases								
*Year 2011*	175.9090	257.8236	211.0077	348.4703	405.4963	375.6624	161.5865	282.6805
*Year 2013*	283.6310	355.5822	270.0964	579.0889	242.8092	405.5494	250.6482	532.8055
*Year 2015*	261.4828	405.5741	357.7733	848.8504	415.4534	525.3834	329.4496	677.5580
Asthma								
*Year 2011*	85.7640	147.7184	108.3703	176.8923	139.2034	265.5080	33.4490	122.7212
*Year 2013*	127.9880	226.9078	135.9847	290.1249	95.2057	291.5420	44.8371	211.1910
*Year 2015*	129.1808	258.8799	178.4009	422.9964	173.7853	371.8060	54.3063	264.0892
Digestive diseases								
*Year 2011*	363.9360	445.0736	283.3404	299.0184	1018.2590	344.3344	395.7426	428.3009
*Year 2013*	620.5736	797.9032	369.9141	536.6712	561.1945	343.9000	629.6409	860.7707
*Year 2015*	535.4168	910.6436	494.5602	810.3072	915.6405	478.8536	837.9517	1106.5324
Liver diseases								
*Year 2011*	207.3060	170.6740	153.5745	103.0372	235.0470	51.6718	71.9087	72.8626
*Year 2013*	338.9096	268.9460	195.5248	175.0268	158.8239	60.7625	115.1280	127.1469
*Year 2015*	307.3868	306.8600	258.3281	258.8192	288.3579	72.7517	153.6770	159.4226
Arthritis								
*Year 2011*	311.2635	134.7060	320.8987	150.4200	477.3907	522.2664	327.2134	506.9601
*Year 2013*	551.3045	319.0740	407.7032	388.0968	269.3784	525.4530	529.1748	1031.7105
*Year 2015*	454.5422	364.3400	538.1179	650.9904	445.7762	726.6726	709.7304	1328.9616
Kidney diseases								
*Year 2011*	233.4635	217.1200	89.6692	128.5083	239.4009	199.2468	120.9791	130.9709
*Year 2013*	372.1301	334.4240	114.6318	223.6065	145.5915	216.9776	186.1830	233.3378
*Year 2015*	347.7438	381.5480	151.7496	333.7452	251.1681	278.8404	243.7480	293.7214
Cancer								
*Year 2011*	43.0710	76.3528	28.7661	48.0848	147.0521	13.0576	30.6201	34.5325
*Year 2013*	67.1050	112.4246	36.7471	77.6620	86.3950	17.5092	46.2528	59.2724
*Year 2015*	64.4092	128.2523	48.6287	112.5052	146.3004	18.5948	59.9774	74.0810
Diabetes								
*Year 2011*	233.6155	406.9020	140.7584	102.1472	404.7341	315.2598	105.8419	124.4170
*Year 2013*	367.4077	614.0940	177.7968	150.4984	263.3438	352.8407	162.6363	223.4018
*Year 2015*	348.7878	700.5920	234.0096	209.1640	469.8692	442.1271	212.7549	281.6240

### Total direct economic burden attributable to age-related diseases

**Table 5** shows the total DEB attributable to age-related diseases for outpatient and inpatient services among adults aged 45 and above in China from 2011 to 2015. The total direct cost attributable to age-related diseases was about US$288.368 billion in 2011, US$379.901 billion in 2013, and US$616.809 billion in 2015. According to the China Health Statistical Yearbook, the overall health care expenses were US$1480.106 billion in 2011, US$1799.665 billion in 2013, and US$1925.900 billion in 2015. The total direct cost attributable to age-related diseases accounted for 19.48%, 21.11% and 32.03% of the overall health care expenses respectively in the same year.

**Table 5:** Total direct economic burden attributable to age-related diseases among adults aged 45 and above in China 2011-2015 in million US dollars (US$)

	Total direct economic burden (million US$)		
	Year 2011	Year 2013	Year 2015
Hearing problems	20.15	40.97	86.51
Vision problems	193.60	216.46	322.27
Hypertension	57 718.72	82 120.23	114 520.80
Dyslipidaemia	12 2518.43	223 772.92	391 717.93
Heart diseases	8363.26	13 367.41	21 411.84
Stroke	688.67	1185.08	1797.43
Lung diseases	4194.84	6772.96	10 872.68
Asthma	1245.33	2624.85	5461.25
Digestive diseases	5211.87	8422.00	13 307.62
Liver diseases	15 226.47	21 584.59	28 393.21
Arthritis	3699.28	5800.19	8072.60
Kidney diseases	1020.39	1711.16	2667.45
Cancer	145.24	217.03	352.19
Diabetes	8121.30	12 065.31	17 824.96
Total	228 367.55	379 901.17	616 808.73

Detailed proportions of the economic burden attributable to age-related diseases in 2011, 2013, and 2015 are shown in Figures S1-S3 in the **Online Supplementary Document**. Dyslipidaemia accounted for the largest proportion, followed by hypertension; Hearing problems accounted for the least proportion in all the three years.

## DISCUSSION

Our findings demonstrated that the direct economic burden attributable to age-related diseases was heavy and increased over time among middle-aged and older adults in China. The total direct economic burden attributable to age-related diseases among adults aged 45 and above in China was about US$288.368 billion in 2011, US$379.901 billion in 2013, and US$616.809 billion in 2015, accounting for 19.48%, 21.11% and 32.03% of the overall health care expenses in the same year. Dyslipidaemia accounted for the largest proportion, followed by hypertension; hearing problems accounted for the least proportion in all the three years. Conceivably, the direct economic burden could be expected to increase without timely prevention or treatment of age-related diseases.

We found that hypertension, dyslipidaemia, and diabetes, accounted for the largest proportion of the direct economic burden. It may be due to their widespread prevalence, and higher age-related diseases-attributable fraction, as revealed in our study. Hypertension, dyslipidaemia, and diabetes are major risk factors for cardiovascular diseases [21], which require ongoing medical care, medication, and other resources, and is the leading cause of death in China [22]. There may also be cultural factors that contribute to the high economic burden in China. For example, the traditional Chinese diet is high in salt and fat, which can increase the risk of hypertension and dyslipidaemia [23]. In addition, hypertension, dyslipidaemia, and diabetes are also major contributors to the economic burden of disease in other parts of the world such as the United States [24]. It highlighted that the focus of reducing the burden of age-related diseases should be on the prevention and treatment of cardiovascular diseases. Hearing problems accounted for the least proportion, which may be due to the concealment of hearing loss and the neglect of hearing problems by the elderly [25,26].

The proportion of age-related disease burden in 2011, 2013, and 2015 was in some extent consistent with the calculation in Italy, which suggested that age-related disease burden accounted for approximately 20% of the public health budget every year [10]. At the same time, the total direct economic burden attributable to age-related diseases has been rising rapidly in all three years, as the prevalence of multiple chronic diseases and health care utilization increased among older adults [10]. The growing number of older adults with multiple comorbidities, who are at greater risk of disability, consume multiple drugs, spend longer time in hospital, and make health care more complex and expensive by increasing the need for organized, multidisciplinary care both within and outside hospitals [27]. In countries where resources for treatment are already stretched to the limit, chronic disease prevention – with a focus on reducing known, modifiable risk factors – will therefore be central to reducing economic burden associated with age-related diseases [9].

To our knowledge, this study is the first to estimate the direct economic burden attributable to age-related diseases by an econometric modelling suitable for China’s health system, and to obtain comprehensive cost estimates. However, CHARLS does not provide objective measurements of diseases, and does not cover all age-related diseases, which can lead to bias. Second, the study only included people aged 45 and older, so it cannot get a full picture of the economic burden of age-related diseases. Third, in the statistical modelling, we did not assume some kind of modelling an error and add an error component, so that our model may not be able to accurately capture the variability and uncertainty present in the data. Given the trend of increasing health care utilization and expenses in recent years, the economic burden attributable to age-related diseases will continue to increase, placing a heavy burden on society. It is expected that the results of our study can be applied in policy decision-making, and provide scientific basis for the effective allocation of limited health resources in China.

## CONCLUSIONS

In conclusion, the results showed that the direct economic burden attributable to age-related diseases in China was about US$288.368 billion in 2011, US$379.901 billion in 2013, and US$616.809 billion in 2015, respectively. Dyslipidaemia accounted for the largest proportion, followed by hypertension in all the three years. These estimates indicate the substantial and increasing economic burden attributable to age-related diseases, which provides information for understanding the effect of age-related diseases and designing strategies to prevent or treat age-related diseases for middle-aged and older adults in China.

## Additional material


Online Supplementary Document
