# Supplementary material for: Direct economic burden attributable to age-related diseases in China: An econometric modelling study
Source: J Glob Health. 2023 May 5;13:04042. doi: 10.7189/jogh.13.04042 (PMC10164278; doi:10.7189/jogh.13.04042)

**Online Supplementary Document**

**Title: Direct economic burden attributable to age-related diseases in China: An econometric modelling study**

**Authors:** Xin Ye, Ming Wang, Yiqi Xia, Ping He, Xiaoying Zheng

## Appendix

**Contents**

**Supplementary Table……………………………………………..….2**

**Supplementary Figures…………………………………………………4**

# Supplementary Table

## Table S1. Summary statistics of age-related diseases, 2011–2015 (N = 32,418).

| Characteristics | The whole sample |
|---|---|
| **Hearing problems** | |
| Yes | 6,002 (18.51%) |
| No | 26,416 (81.49%) |
| **Vision problems** | |
| Yes | 1,655 (5.35%) |
| No | 29,287 (94.65%) |
| **Hypertension** | |
| Yes | 9,916 (30.96%) |
| No | 22,117 (69.04%) |
| **Dyslipidemia** | |
| Yes | 4,485 (14.28%) |
| No | 26,919 (85.72%) |
| **Heart diseases** | |
| Yes | 5,103 (15.91%) |
| No | 26,964 (84.09%) |
| **Stroke** | |
| Yes | 1,022 (3.17%) |
| No | 31,226 (96.83%) |
| **Lung diseases** | |
| Yes | 4,125 (12.82%) |
| No | 28,052 (87.18%) |
| **Asthma** | |
| Yes | 1,781 (5.53%) |
| No | 30,419 (94.47%) |
| **Digestive diseases** | |
| Yes | 9,087 (28.24%) |
| No | 23,088 (71.76%) |
| **Liver diseases** | |
| Yes | 1,754 (5.47%) |
| No | 30,289 (94.53%) |
| **Arthritis** | |
| Yes | 13,380 (41.57%) |
| No | 18,808 (58.43%) |
| **Kidney diseases** | |
| Yes | 2,660 (8.30%) |
| No | 29,382 (91.70%) |
| **Cancer** | |
| Yes | 442 (1.37%) |
| No | 31,709 (98.63%) |

| | |
|---|---|
| **Diabetes** | |
| Yes | 2,694 (8.44%) |
| No | 29,209 (91.56%) |

*Note:* SD – standard deviation; ADLs – activities of daily living; IADLs – instrumental activities of daily living

## Supplementary Figures

**Supplemental Figure 1.** Proportions of total direct economic burden attributable to age-related diseases among adults aged 45 and above in China in 2011.

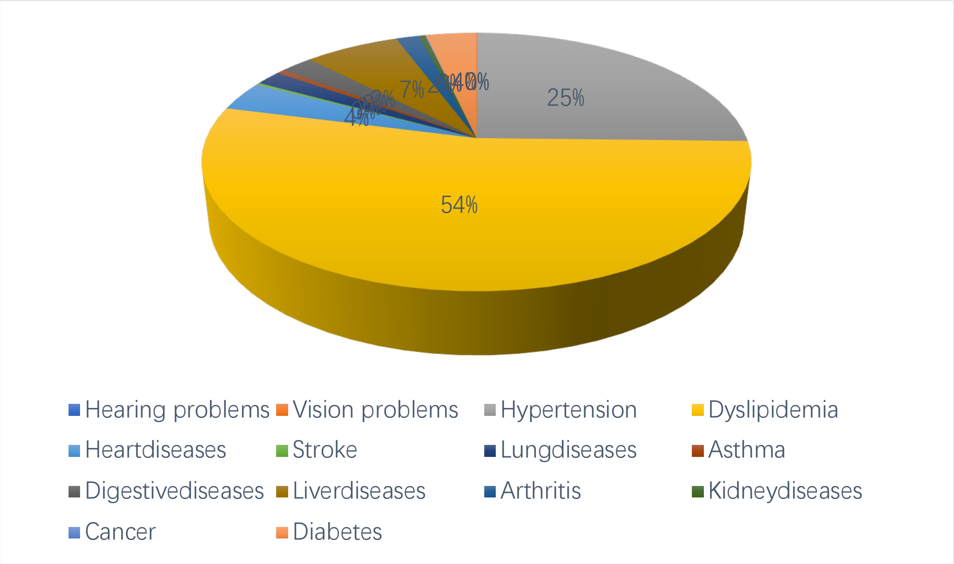

**Supplemental Figure 2.** Proportions of total direct economic burden attributable to age-related diseases among adults aged 45 and above in China in 2013.

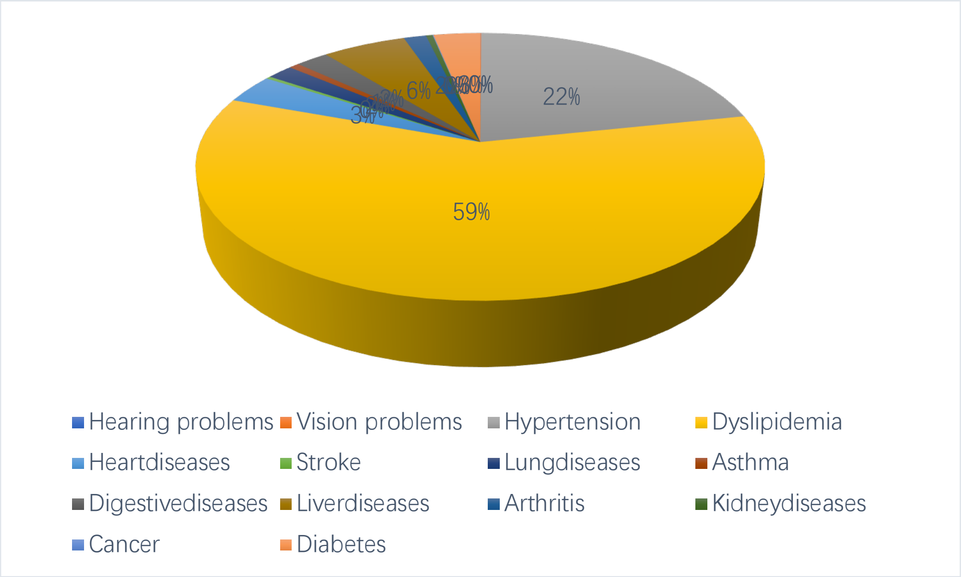

**Supplemental Figure 3.** Proportions of total direct economic burden attributable to age-related diseases among adults aged 45 and above in China in 2015.

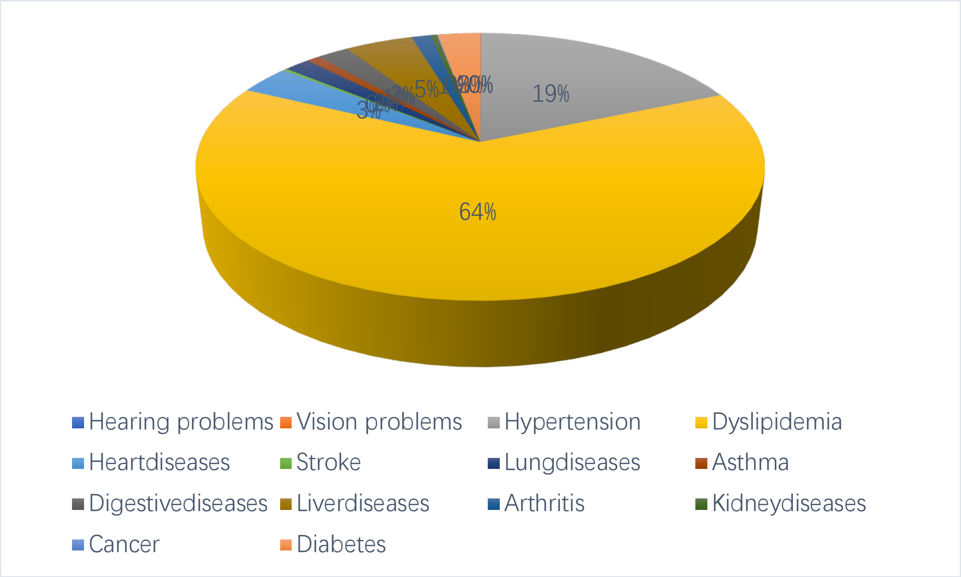

Supplement: Online Supplementary Document [file jogh-13-04042-s001.pdf]
